# COVID-19 and Post-Acute COVID-19 Syndrome: From Pathophysiology to Novel Translational Applications

**DOI:** 10.3390/biomedicines10010047

**Published:** 2021-12-27

**Authors:** Pasquale Ambrosino, Anna Lanzillo, Mauro Maniscalco

**Affiliations:** 1 Istituti Clinici Scientifici Maugeri IRCCS, Cardiac Rehabilitation Unit of Telese Terme Institute, 82037 Telese Terme, Italy; pasquale.ambrosino@icsmaugeri.it; 2 Istituti Clinici Scientifici Maugeri IRCCS, Neurological Rehabilitation Unit of Telese Terme Institute, 82037 Telese Terme, Italy; anna.lanzillo@icsmaugeri.it; 3 Istituti Clinici Scientifici Maugeri IRCCS, Pulmonary Rehabilitation Unit of Telese Terme Institute, 82037 Telese Terme, Italy

In late 2019, the novel severe acute respiratory syndrome coronavirus 2 (SARS-CoV-2) gave rise to a public health emergency, culminating in the declaration of a pandemic in March 2020. SARS-CoV-2 can determine coronavirus disease 2019 (COVID-19), which ranges from a flu-like illness to a serious life-threatening condition requiring hospitalization in intensive care units (ICU) [1]. Moreover, the evidence of pulmonary abnormalities and long-term cardiovascular complications in COVID-19 survivors suggests the presence of a post-acute COVID-19 syndrome and residual disability, with the need for rehabilitation strategies in a substantial proportion of convalescent patients [2]. This is consistent with the finding of muscular weakness, physical deconditioning, psychological distress, cognitive impairment, and a number of other functional disorders even months after a negative swab test [3].

Growing evidence suggests a central role of endothelial dysfunction and systemic inflammation in the pathogenesis of most COVID-19 manifestations [4]. Accordingly, persistent endothelial dysfunction and a prolonged inflammatory response have been documented even after the acute phase [5]. However, the pathophysiological mechanisms determining the acute and post-acute manifestations of COVID-19 are still a matter requiring study, given that a better understanding of these mechanisms would allow for the implementation of more effective personalized approaches.

In this Special Issue, we aimed to collect a number of original research articles and reviews on the pathophysiology and diagnostics of COVID-19 and post-acute COVID-19 syndrome, with a focus on new prognostic and therapeutic applications.

Thus, some high-quality reviews focusing on different aspects of the complex interplay between endothelial dysfunction, inflammation, and epigenetics were published. Yan et al. provided an interesting overview of the systemic effects of the post-acute COVID-19 syndrome, emphasizing the importance of exercise-based, personalized multidisciplinary rehabilitation strategies [6]. The epigenetic mechanisms potentially underlying the pathogenesis of COVID-19 were analyzed in another article, with particular regard to the involvement of epigenetic factors such as histone deacetylase in angiotensin-converting enzyme 2 (ACE2) expression [7]. Karn et al. discussed the promising role of extracellular vesicle-based therapy for COVID-19, with a focus on the ongoing clinical trials and potential limitations of such a therapeutic approach [8]. An interesting meta-analysis was also published in this Special Issue. In their study, the authors provided a collection of information on the prevalence of some manifestations of the post-acute COVID-19 syndrome, including computed tomography (CT) abnormalities, impaired lung function, fatigue, dyspnea, chest pain, cough, and quality of life impairment [9]. Overall, they concluded that the risk of persistent manifestations and residual disability at a 3-month follow-up is not negligible, suggesting long-term monitoring of these patients to promptly predict and manage potential life-threatening complications.

A number of preclinical, clinical, and functional studies also fell within the scope of this Special Issue. In a noteworthy article by Berezina et al. [10], the authors investigated the role of the indicators of biological and subjective aging in COVID-19 patients. Interestingly, a combination of these indicators was found to be a risk factor for severe forms of the disease. In another study on a retrospective cohort of 2040 COVID-19 patients [11], the prognostic ability of the pulse oximetry saturation/fraction of inspired oxygen ratio (SpO_2_/FiO_2_) and the ratio of SpO_2_/FiO_2_ to the respiratory rate (ROX index) was investigated. The SpO_2_/FiO_2_ ratio was found to be better than the ROX index in predicting the risk of invasive mechanical ventilation (IMV), with an area under the receiver operating characteristic (ROC) curve of 0.801 and 0.725, respectively. Considering an IMV rate of 10.1% in their study population, the authors concluded that the SpO_2_/FiO_2_ ratio may present a cost-effective tool in this emergency scenario. The innate inflammatory response also showed a good prognostic value in terms of ICU admittance and death in a series of 287 COVID-19 patients [12]. In a further clinical study [13], a total of 133 convalescent COVID-19 patients showed significantly lower values of endothelium-dependent flow-mediated dilation (FMD) compared to age- and sex-matched controls. Moreover, a direct and persistent correlation between the severity of pulmonary and vascular disease was reported, providing preliminary information on the potential usefulness of exercise-based rehabilitation and strategies targeting endothelial function. The central role of the endothelial dysfunction in the pathogenesis of the disease was confirmed also in cell cultures of human lung microvascular endothelial cells (HLMVEC), suggesting that pro-inflammatory mediators released by spike-activated macrophages may amplify the activation of endothelial cells [14]. One of the most interesting reports of this Special Issue was on the molecular interactions between the viral spike protein and the host angiotensin-converting enzyme 2 (ACE2) protein, with a comparison between the previous severe acute respiratory syndrome coronavirus 1 (SARS-CoV-1) and the novel coronavirus [15]. In their in silico analysis, the authors evidenced some peculiarities in the interactions of the two spike-ACE2 complexes, with a key role of R426 and K417 amino acid residues in the receptor-binding domains of SARS-CoV-1 and SARS-CoV-2, respectively. Other authors [16,17] have investigated the potential therapeutic role of enisamium and hydrogen sulfide in culture models, given their ability to inhibit viral RNA synthesis or virus entry into epithelial cells, respectively. Azgomi et al. [18] proposed instead a rapid and simple multiparameter assay to quantify spike-specific CD4^+^ and CD8^+^ T-cells after SARS-CoV-2 vaccination in both healthy donors and subjects with B-cell compart impairment. Most importantly, the authors showed that vaccination with the mRNA vaccine BNT162b2 gives a robust cell-mediated immunological memory against spike protein antigens, independently from the titer of neutralizing antibodies. This may have relevant clinical implications, since the vaccine-induced T-cell response against SARS-CoV-2 can develop independently from B-cells. Vaccine-induced changes were studied in another interesting article by Colarusso et al. [19], aimed at identifying blood biomarkers potentially predicting the development of lung fibrotic changes in post-acute COVID-19 patients. The authors found that C-reactive protein, C5b-9 complement complex, lactate dehydrogenase, but not interleukin-6 were higher in COVID-19 survivors than in non-COVID-19 vaccinated participants. Interestingly, COVID-19 patients with residual ground-glass opacities at CT exhibited higher plasma levels of interleukin-1α and transforming growth factor-β when compared to both patients without lung abnormalities and vaccinated subjects, with lower plasma levels of interferon-γ.

Other studies [20] have been published in this Special Issue, including some relevant case reports [21,22], all contributing to provide a stimulating insight into the pathophysiology and diagnostics of COVID-19 and post-acute COVID-19 syndrome. Some prognostic and therapeutic applications have been analyzed in both review and original articles, with interesting new findings and potentially relevant repercussions in clinical practice and future research. Meanwhile, further laboratory and translational research is needed to better understand the molecular mechanisms of COVID-19 both in the acute phase and during convalescence. This could help establish comprehensive and personalized prevention, interventional and rehabilitation strategies aimed at reducing disease progression, long-term complications, occupational disability, and quality of life impairment.

## Data Availability

No datasets were generated or analysed during the current study.

## References

[1] Singh S., McNab C., Olson R.M., Bristol N., Nolan C., Bergstrom E., Bartos M., Mabuchi S., Panjabi R., Karan A. (2021). How an outbreak became a pandemic: A chronological analysis of crucial junctures and international obligations in the early months of the COVID-19 pandemic. Lancet.

[2] Ambrosino P., Papa A., Maniscalco M., Di Minno M.N.D. (2021). COVID-19 and functional disability: Current insights and rehabilitation strategies. Postgrad. Med. J..

[3] Gloeckl R., Leitl D., Jarosch I., Schneeberger T., Nell C., Stenzel N., Vogelmeier C.F., Kenn K., Koczulla A.R. (2021). Benefits of pulmonary rehabilitation in COVID-19: A prospective observational cohort study. ERJ Open Res..

[4] Evans P.C., Rainger G.E., Mason J.C., Guzik T.J., Osto E., Stamataki Z., Neil D., Hoefer I.E., Fragiadaki M., Waltenberger J. (2020). Endothelial dysfunction in COVID-19: A position paper of the ESC Working Group for Atherosclerosis and Vascular Biology, and the ESC Council of Basic Cardiovascular Science. Cardiovasc. Res..

[5] Ambrosino P., Molino A., Calcaterra I., Formisano R., Stufano S., Spedicato G.A., Motta A., Papa A., Di Minno M.N.D., Maniscalco M. (2021). Clinical Assessment of Endothelial Function in Convalescent COVID-19 Patients Undergoing Multidisciplinary Pulmonary Rehabilitation. Biomedicines.

[6] Yan Z., Yang M., Lai C.L. (2021). Long COVID-19 Syndrome: A Comprehensive Review of Its Effect on Various Organ Systems and Recommendation on Rehabilitation Plans. Biomedicines.

[7] Kaneko S., Takasawa K., Asada K., Shinkai N., Bolatkan A., Yamada M., Takahashi S., Machino H., Kobayashi K., Komatsu M. (2021). Epigenetic Mechanisms Underlying COVID-19 Pathogenesis. Biomedicines.

[8] Karn V., Ahmed S., Tsai L.W., Dubey R., Ojha S., Singh H.N., Kumar M., Gupta P.K., Sadhu S., Jha N.K. (2021). Extracellular Vesicle-Based Therapy for COVID-19: Promises, Challenges and Future Prospects. Biomedicines.

[9] Sanchez-Ramirez D.C., Normand K., Zhaoyun Y., Torres-Castro R. (2021). Long-Term Impact of COVID-19: A Systematic Review of the Literature and Meta-Analysis. Biomedicines.

[10] Berezina T.N., Rybtsov S. (2021). Acceleration of Biological Aging and Underestimation of Subjective Age Are Risk Factors for Severe COVID-19. Biomedicines.

[11] Alberdi-Iglesias A., Martin-Rodriguez F., Ortega Rabbione G., Rubio-Babiano A.I., Nunez-Toste M.G., Sanz-Garcia A., Del Pozo Vegas C., Castro Villamor M.A., Martin-Conty J.L., Jorge-Soto C. (2021). Role of SpO_2_/FiO_2_ Ratio and ROX Index in Predicting Early Invasive Mechanical Ventilation in COVID-19. A Pragmatic, Retrospective, Multi-Center Study. Biomedicines.

[12] Monserrat J., Asunsolo A., Gomez-Lahoz A., Ortega M.A., Gasalla J.M., Gasulla O., Fortuny-Profitos J., Mazaira-Font F.A., Teixido Roman M., Arranz A. (2021). Impact of the Innate Inflammatory Response on ICU Admission and Death in Hospitalized Patients with COVID-19. Biomedicines.

[13] Ambrosino P., Calcaterra I., Molino A., Moretta P., Lupoli R., Spedicato G.A., Papa A., Motta A., Maniscalco M., Di Minno M.N.D. (2021). Persistent Endothelial Dysfunction in Post-Acute COVID-19 Syndrome: A Case-Control Study. Biomedicines.

[14] Rotoli B.M., Barilli A., Visigalli R., Ferrari F., Dall’Asta V. (2021). Endothelial Cell Activation by SARS-CoV-2 Spike S1 Protein: A Crosstalk between Endothelium and Innate Immune Cells. Biomedicines.

[15] Giordano D., De Masi L., Argenio M.A., Facchiano A. (2021). Structural Dissection of Viral Spike-Protein Binding of SARS-CoV-2 and SARS-CoV-1 to the Human Angiotensin-Converting Enzyme 2 (ACE2) as Cellular Receptor. Biomedicines.

[16] Elli S., Bojkova D., Bechtel M., Vial T., Boltz D., Muzzio M., Peng X., Sala F., Cosentino C., Goy A. (2021). Enisamium Inhibits SARS-CoV-2 RNA Synthesis. Biomedicines.

[17] Pozzi G., Masselli E., Gobbi G., Mirandola P., Taborda-Barata L., Ampollini L., Carbognani P., Micheloni C., Corazza F., Galli D. (2021). Hydrogen Sulfide Inhibits TMPRSS2 in Human Airway Epithelial Cells: Implications for SARS-CoV-2 Infection. Biomedicines.

[18] Azgomi M.S., La Manna M.P., Badami G.D., Ragonese P., Trizzino A., Dieli F., Caccamo N. (2021). A Rapid and Simple Multiparameter Assay to Quantify Spike-Specific CD4 and CD8 T Cells after SARS-CoV-2 Vaccination: A Preliminary Report. Biomedicines.

[19] Colarusso C., Maglio A., Terlizzi M., Vitale C., Molino A., Pinto A., Vatrella A., Sorrentino R. (2021). Post-COVID-19 Patients Who Develop Lung Fibrotic-like Changes Have Lower Circulating Levels of IFN-β but Higher Levels of IL-1α and TGF-β. Biomedicines.

[20] Kaziród-Wolski K., Sielski J., Sidło J., Januszek R., Siudak Z. (2021). The Most Relevant Factors Affecting the Perioperative Death Rate in Patients with Acute Coronary Syndrome and COVID-19, Based on Annual Follow-Up in the ORPKI Registry. Biomedicines.

[21] Agarwal R.N., Aggarwal H., Verma A., Tripathi M.K. (2021). A Case Report of a Patient on Therapeutic Warfarin Who Died of COVID-19 Infection with a Sudden Rise in D-Dimer. Biomedicines.

[22] Ciolac D., Racila R., Duarte C., Vasilieva M., Manea D., Gorincioi N., Condrea A., Crivorucica I., Zota E., Efremova D. (2021). Clinical and Radiological Deterioration in a Case of Creutzfeldt-Jakob Disease following SARS-CoV-2 Infection: Hints to Accelerated Age-Dependent Neurodegeneration. Biomedicines.

